# Enhancing Lessons on the Internet of Things in Science, Technology, Engineering, and Medical Education with a Remote Lab

**DOI:** 10.3390/s24196424

**Published:** 2024-10-04

**Authors:** Sofia Amador Nelke, Dan Kohen-Vacs, Michael Khomyakov, Maria Rosienkiewicz, Joanna Helman, Mariusz Cholewa, Mateusz Molasy, Anna Górecka, José-Francisco Gómez-González, Maxime Bourgain, Athith Sagar, Giovanni Berselli, Daniel Blank, Michael Winokur, Arriel Benis

**Affiliations:** 1 Faculty of Industrial Engineering and Technology Management, Holon Institute of Technology, Holon 5810201, Israel; sofiamador@gmail.com (S.A.N.); michael.khomyakov@qinflow.com (M.K.); michaelw@hit.ac.il (M.W.); 2 Information System Program, School of Science and Technology, Ono Academic College, Kiryat Ono 5500000, Israel; 3 Faculty of Instructional Technologies, Holon Institute of Technology, Holon 5810201, Israel; mrkohen@hit.ac.il; 4 Department of Laser Technologies, Automation and Production Management, Wroclaw University of Science and Technology, Lukasiewicza 5, 50-370 Wroclaw, Poland; maria.rosienkiewicz@pwr.edu.pl (M.R.); joanna.helman@pwr.edu.pl (J.H.); mariusz.cholewa@pwr.edu.pl (M.C.); mateusz.molasy@pwr.edu.pl (M.M.); anna.gorecka@pwr.edu.pl (A.G.); 5 Institute of Technology Transfer Sp. z o.o., Na Grobli 15, 50-421 Wroclaw, Poland; 6 Departamento de Ingeniería Industrial, Escuela Superior de Ingeniería y Tecnología, Universidad de La Laguna, 30206 San Cristóbal de La Laguna, Spain; jfcgomez@ull.edu.es; 7 EPF—Graduate School of Engineering, 55 Avenue du Président Wilson, 94230 Cachan, France; maxime.bourgain@epf.fr; 8 Institut de Biomécanique Humaine Georges Charpak, Arts et Métiers Institute of Technology, IBHGC, 75013 Paris, France; 9 Centria University of Applied Sciences, Vierimaantie 7, 84100 Ylivieska, Finland; athith.sagar@centria.fi; 10 Department of Mechanical Engineering, Energetics, Management and Transportation, University of Genoa, Via all’Opera Pia 15/A, 16145 Genova, Italy; giovanni.berselli@unige.it; 11 Department of Advanced Robotics, Istituto Italiano di Tecnologia, Via S. Quirico 19d, 16163 Genova, Italy; 12 Department of Information, Universidad Blas Pascal, Córdoba 5147, Argentina; blankjdaniel@gmail.com; 13 Department of Digital Medical Technologies, Holon Institute of Technology, Holon 5810201, Israel

**Keywords:** remote laboratory, technology-enhanced learning, internet of things, interactive learning, project-based learning, education, educational technology, generative artificial intelligence, hands-on learning, formative feedback, user–computer interface

## Abstract

Integrating remote Internet of Things (IoT) laboratories into project-based learning (PBL) in higher education institutions (HEIs) while exploiting the approach of technology-enhanced learning (TEL) is a challenging yet pivotal endeavor. Our proposed approach enables students to interact with an IoT-equipped lab locally and remotely, thereby bridging theoretical knowledge with practical application, creating a more immersive, adaptable, and effective learning experience. This study underscores the significance of combining hardware, software, and coding skills in PBL, emphasizing how IoTRemoteLab (the remote lab we developed) supports a customized educational experience that promotes innovation and safety. Moreover, we explore the potential of IoTRemoteLab as a TEL, facilitating and supporting the understanding and definition of the requirements of remote learning. Furthermore, we demonstrate how we incorporate generative artificial intelligence into IoTRemoteLab’s settings, enabling personalized recommendations for students leveraging the lab locally or remotely. Our approach serves as a model for educators and researchers aiming to equip students with essential skills for the digital age while addressing broader issues related to access, engagement, and sustainability in HEIs. The practical findings following an in-class experiment reinforce the value of IoTRemoteLab and its features in preparing students for future technological demands and fostering a more inclusive, safe, and effective educational environment.

## 1. Introduction

### 1.1. Background

The Internet of Things (IoT) is rapidly becoming a significant part of daily life across “21st-century” industries. By combining software, hardware, and interactions between different kinds of components over networks, IoT allows the manipulation of physical objects within remote environments. Ashton defined IoT as “a system where the Internet is connected to the physical world via ubiquitous sensors” [1,2]. This general definition of the 2000s is adapted to different technological evolutions [3]. Hence, sectors such as industrial manufacturing and automation, healthcare and wellness, and education and training develop and offer IoT-based products, which provide opportunities to exercise project-based learning (PBL) in realistic settings [4,5,6,7].

Integrating IoT into educational settings aligns with the technology-enhanced learning (TEL) principles, which seek to improve the accessibility, effectiveness, and engagement of learning experiences through digital tools and innovative practices [8,9,10]. Within this context, TEL leverages the potential of remote labs [11,12] defined as enhancing the accessibility of experimental setups, offering a distance teaching framework meeting the learner’s hands-on needs [13,14]. In this sense, this deployment can enhance both teaching and learning in various realistic contexts and settings aligned with the requirements of the mentioned sectors of modern industries. By incorporating online platforms, TEL creates a versatile, inclusive, and practical learning environment that bridges the gap between theoretical knowledge and practical application, making educational experiences more engaging across various disciplines [12,13,14,15].

Nowadays, higher education institutions (HEIs) increasingly adopt IoT remote labs, particularly in response to global challenges that impose restrictions like those experienced during the COVID-19 pandemic [12,15]. This trend offers hands-on experiences despite physical constraints [16,17]. Thus, labs that integrate IoT capabilities ensure learning continuity while a remote working mode is required or enforced. In such cases, remote labs enable teaching and learning continuity, as well as continuous collaboration between industry and academic partners [16,18,19,20]. Moreover, from a TEL perspective, AI allows personalizing the learning process to the educational requirements, which is crucial. Nowadays, embedding AI capabilities in these systems is emerging along the student’s learning pathway [21,22,23,24,25,26]. However, this integration also presents risks, such as over-reliance on technology, biases in AI algorithms, and privacy concerns, necessitating ongoing evaluation and adaptation to ensure educational outcomes meet evolving industry standards [27,28,29,30]. Consequently, addressing these challenges proactively must lead to significantly enhancing the quality and accessibility of remote-based education, preparing students for the demands of the 21st-century workforce [31,32].

Sustainability is also a critical decision factor in implementing remote labs as real-world-oriented infrastructures, facilitating access to training and experimentation through safe digital remote trials, even with expensive or hazardous equipment, but without the need for many replicates to be used simultaneously. This transformation in education and training enhances scientific and technical literacy in under-resourced areas and helps maintain professional abilities and skills required in professional practice [33,34].

### 1.2. Aims, Objectives, and Goals

We aim to develop a platform facilitating “Science, Technology, Engineering, and Medical” (STEM) education enhanced with remote labs. This leads to an educational approach that utilizes TEL to facilitate project-based learning (PBL) activities, specifically tailored to the needs of innovative curricula in STEM, aligning with the requirements of the realistic settings of modern professional practice crossover industries. Through this integration, we anticipate enhanced practical skills and better preparation among students to meet the job market challenges. In the present study, we focused our efforts on undergraduate students in “Industrial Engineering and Technology Management”, looking at this field as multidisciplinary, preparing students to work at different positions in all the business and industry fields.

Below, we address the deployed outcome of this research project, ”IoTRemoteLab”. Our main objective is to facilitate their multidisciplinary education while maintaining an efficient, appealing, accessible, and inclusive learning process, allowing them to access and control lab equipment remotely while maintaining an efficient, appealing, accessible, and inclusive learning process and exploiting IoTRemoteLab’s features.

Our primary goal is to demonstrate the effectiveness of TEL in supporting a low-cost, user-friendly PBL process, resulting in an educational process that is both effective and appealing. In this study, we focused on leveraging TEL to enhance students’ experience while using the lab as part of a PBL process [16,35]. This approach allows the educational content’s tailoring, enabling IoTRemoteLab‘s adoption, adaptation, customization, and exploitation to provide personalized learning experiences.

IoTRemoteLab’s design, development, and deployment were based on a comprehensive educational and technological discovery requirements process [36]. In the second step, we defined its architecture before implementing it physically with related software. The third step consisted of evaluating IoTRemoteLab with students involved in an IoT course. Then, we discussed IoTRemoteLab’s current strengths and limitations before considering potential future perspectives.

## 2. From Techno-Educational Requirements towards Deployment of Lab

A clear understanding of educational and technological requirements is critical to fit the needs of remote lab-based courses. Thus, the techno-educational requirement’s discovery step started with semi-structured interviews with lecturers, enabling IoTRemoteLab’s initial design and development. Specifically, the lecturers provided insights into the challenges and opportunities within their teaching practices in the context of labs, enabling local and remote teaching and learning practices. We followed this step with another evaluation involving undergraduate students using the lab during its initial implementation.

The insights from all these interviews (both of lecturers and students) were used in IoTRemoteLab’s architecture design and development. Then, students were also required to answer a questionnaire focusing on various aspects of their experience [16,37].

We designed and spread the questionnaire mentioned above (including 25 items) to assess the students’ satisfaction, perceptions of the educational content, and the effectiveness of hands-on activities. We evaluated other lab aspects, including the support provided by lecturers during its use, its suitability as a learning environment, and its alignment with educational goals. Additionally, students were given the opportunity to provide open feedback on their IoTRemoteLab experiences. The questionnaire featured seven rating questions on a Likert scale from 1 (lowest) to 5 (highest), allowing students to express their level of agreement with various statements. The exploratory nature of this evaluation encouraged participants to offer personal comments, which were crucial for the results’ analysis. This comprehensive feedback process was essential in refining the lab’s design better to meet the needs of both students and educators.

Below, we present the results from the lecturers’ interviews, followed by another presentation of the results expressed by students answering the questionnaire.

We conducted semi-structured interviews with three lectures to enable the initial discovery of requirements for designing and developing IoTRemoteLab. The lecturers relied on automation and robotics, IoT, and digital medical technologies. During these interviews, Lecturers 1 and 2 emphasized the importance of allowing real-time experimentation with IoT and robotic equipment, regardless of the user’s location. This feature is crucial for students, educators, and researchers, as it allows them to conduct experiments remotely while maintaining the experience of a physical lab. This need for flexibility was particularly highlighted by Lecturer 1, who recalled the restrictions imposed during the COVID-19 pandemic, where remote work became essential. IoTRemoteLab should thus accommodate a range of IoT equipment and industrial robots, whether for basic exercises or advanced research projects.

Lecturer 3 highlighted the necessity of supporting asynchronous learning, allowing students to conduct experiments at their own pace and on their own schedule. This flexibility is necessary for accommodating various learning pathways and life circumstances. Additionally, Lecturers 2 and 3 stressed the importance of combining theoretical learning with practical application in a hybrid learning environment. This approach enables students to apply theoretical concepts in real-world settings, reinforcing their understanding and enhancing learning.

The design of the user interface (UI) was another critical consideration. Lecturers 1 and 3 emphasized that the interface should be robust and intuitive, minimizing operational failures and making it easy for users to interact with the equipment. This is particularly important to ensure that students and researchers can focus on their learning or research tasks without being hindered by technical difficulties.

To broaden accessibility, Lecturers 1 and 2 advocated including application programming interfaces (APIs) for embedded systems that do not require coding. This feature would make the lab more accessible to students with diverse technical backgrounds, particularly those focused on health data analytics or other non-technical disciplines. For more advanced users, the lab should also support remote coding and the ability to download and test different scenarios.

Regarding future scalability and adaptability, Lecturers 1 and 3 suggested that the lab should facilitate the easy addition of new equipment as it becomes available, supporting the development of complex systems for advanced academic projects. Future enhancements could include connectivity to cloud and web systems, enabling advanced analytics and integration with tools related to generative artificial intelligence (GenAI) technologies.

However, challenges were also noted by Lecturer 1, who pointed out that the lab’s effectiveness would heavily depend on the robustness of network connections, both at the lab and user ends. Reliable, high-bandwidth connections are essential to ensure smooth and effective operation. Additionally, Lecturers 1 and 3 mentioned the importance of users’ familiarity with the equipment. Periodic physical access to the lab may be necessary to build this familiarity, especially for students and researchers. They mentioned that incorporating advanced practices such as virtual reality (VR) could help bridge this gap and enhance the overall lab experience for those who are fully remote.

The interviewed lecturers mentioned that the lab should accommodate the emerging needs of both HEIs and industry. They emphasized the need to provide their students with a seamless transition from academia to industry. This is strongly related to the aspirations of HEIs to enhance the attractiveness and effectiveness of their STEM programs, ensuring that graduates are well-prepared for the technological demands of the workforce [38]. They strategically adopt new technologies to keep educational offerings cutting-edge [39]. Globally, the three lecturers agreed that industry partners seek graduates with practical experience with advanced technologies that can quickly adapt to workplace challenges. Their involvement ensures educational programs align with industry needs and produce job-ready graduates. In this sense, Lecture 1 gave an example of the “Deep Tech needs”, including their functionalities (https://synergyplatform.pwr.edu.pl/needs (accessed on 1 October 2024)) developed as part of the DEETECHTIVE project (https://www.deetechtive.eu/; https://eit-hei.eu/projects/deetechtive/ (accessed on 1 October 2024)), wherein the participant’s partners aim to allow the industry to specify the competencies expected from students. This communication helps universities improve study programs and course content accordingly.

## 3. Architecture Definition and Development

Above, we reported the main insights from the interviews, which allowed us to design IoTRemoteLab.

### 3.1. Architecture Definition

**Figure 1:**
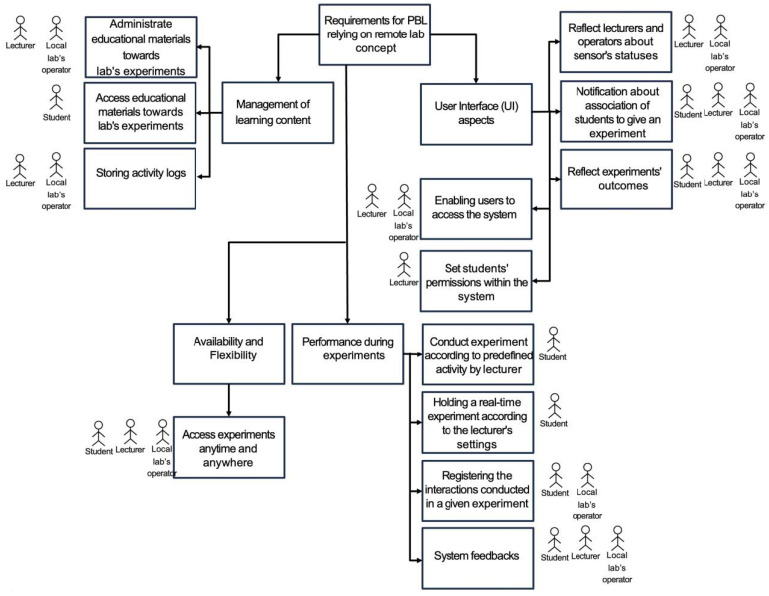
A specification of identified and categorized requirements in our presented efforts.

As illustrated in Figure 1, the requirement specification breaks down overarching needs into specific categories, such as learning content management, IoTRemoteLab availability and flexibility, user performance within the lab, and UI considerations. Hence, a specification is organized in a tree to facilitate organizing IoTRemoteLab’s features for its design and development.

Our process prioritized identifying both technological and educational requirements for deploying a lab enabled by a comprehensive environment that provides a practical and user-friendly experience for students involved in a PBL process in the context of capacity building for the labor market of modern industries.

**Figure 2:**
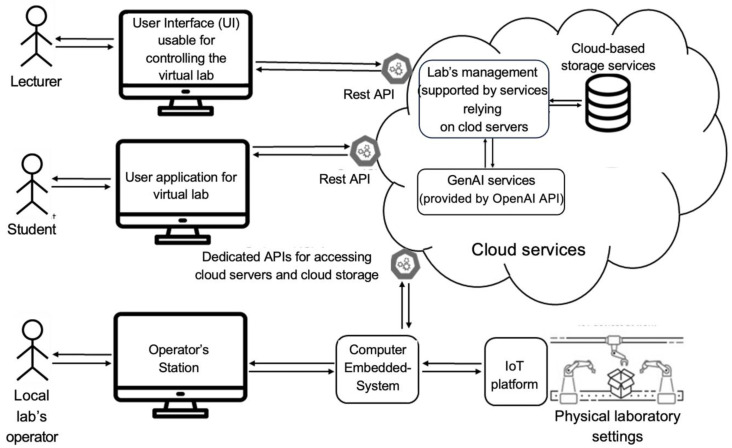
Lab’s architecture.

Based on these requirements, we designed, developed, and deployed a comprehensive architecture of IoTRemoteLab (Figure 2). This last one comprises several critical components for establishing a remote laboratory: (1) a physical lab, (2) an embedded system, and (3) an IoT platform.

### 3.2. Lab’s Development

The general architecture includes a physical laboratory with an experimental setup for studying sensor operations and their applications in automation. The physical part is developed on an Arduino microcontroller (https://www.arduino.cc/ (accessed on 1 October 2024)), which allows connecting to the Internet similarly to a computer and interacting with the lab in real-time.

**Figure 3:**
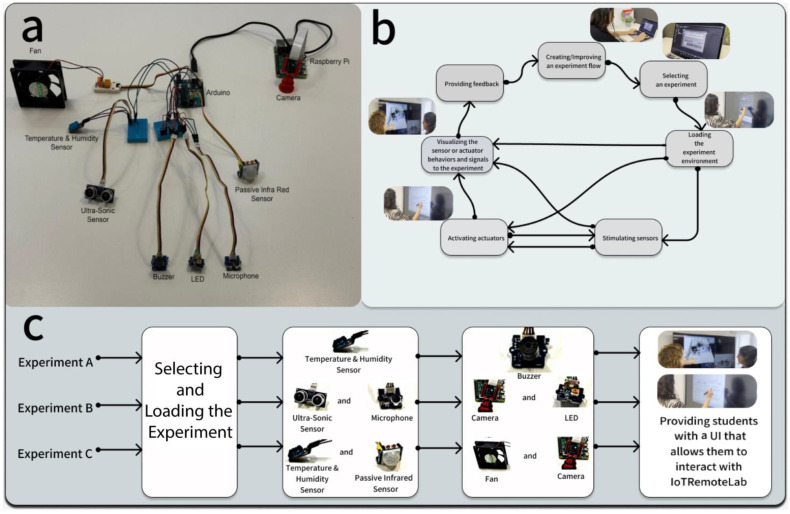
(**a**) An example schematic of a physical implementation of the physical side of the IoTRemoteLab lab comprising temperature/humidity, ultra-sonic (passive infrared), and proximity/distance (ultra-sonic) sensors and actuators such as an LED, a buzzer, and a fan that can be remotely controlled. A camera is integrated both as a sensor capturing pictures from the environment and as an actuator recording while some specific sensors are stimulated. (**b**) Steps in the IoTRemoteLab learning flow showing the whole set of interactions with IoTRemoteLab, from the creation of an experimental exercise by a lecturer to the feedback that the students can submit through the central interactions consisting of understanding how sensors and actuators react when they are stimulated or activated. (**c**) Steps of three experiments (A, B, and C), wherein the students select and load these experiments, and in each one stimulate sensors (A: a temperature and humidity sensor; B: an ultra-sonic sensor and a microphone; C: a temperature and humidity sensor and a passive infrared sensor), activate actuators (A: a buzzer; B: a camera and an LED; C: a fan and a camera), and then visualize on relevant dashboards the results of their actions (stimulating sensors and so activating actuators).

Therefore, IoTRemoteLab has been designed and developed with sensors to support various educational and experimental activities. The key sensors integrated into IoTRemoteLab’s architecture allow measurement of temperature, humidity, pressure, and proximity. These sensors are strategically deployed to monitor and control various parameters in industrial automation scenarios. For instance, temperature and humidity sensors provide real-time data on environmental conditions, which is crucial for experiments related to climate control systems (e.g., electronic or pharmaceutical production lines or warehouses). Pressure sensors measure and regulate fluid dynamics within simulated industrial processes (e.g., refrigerant flow in a cooling system, airflow control in biological safety laboratories investigating pathogenic or infectious organisms posing different hazard levels), or medical devices (e.g., a syringe pump or artificial respirator). In contrast, proximity, movement (such as passive infrared sensors, noted PIR), and light (such as light-dependent resistors, noted LPR) sensors facilitate detecting and measuring distances between objects, enhancing the precision of automation tasks (e.g., presence control, collision detector). Moreover, we have also integrated a microphone to allow the system to react to a basic sound in the current version (e.g., any noise generated by the operator physically present that can be similar to sound instruction like in a smart home). Integrating these sensors within IoTRemoteLab allows students to engage in hands-on experimentation with real-world applications, fostering a deeper understanding of the IoT concepts (Figure 3). This sensor network enhances the functionalities of IoTRemoteLab and supports the development of innovative automation systems, thereby bridging the gap between theoretical knowledge and practical application.

To allow the student to see and understand the effect of actions or environmental changes, we have also included in the current version of IoTRemoteLab a few actuators. Indeed, we integrated an LED that can light and a buzzer that can make a sound, for example, when a preset value is over a threshold defined for one of the sensors (e.g., temperature, humidity, distance); a fan powered on over a preset temperature; a servomotor allowing moving a distance sensor in reaction, for example, to a sound recorded by the microphone (e.g., such as in security systems).

IoTRemoteLab is integrated with an IoT platform (Arduino as pointed out above) that relies on external resources to link the laboratory interface to a controlled cloud environment.

IoTRemoteLab’s Management module is cloud services-based (Figure 2). It is a “hub” responsible for centralizing the managerial aspects of the lab related to various aspects such as the lecturer, student, and operator interactions. Additionally, this module controls the data flow and storage related to IoTRemoteLab’s educational activities organized in a bank of resources (e.g., program codes allowing collecting the measurement of the different sensors, program codes allowing activating actuators) that can be added to new activities or by modifying existing ones. Furthermore, with an experimental feature, we gain insights and recommendations for lecturers and students based on services offered by GenAI (herein provided by OpenAI API, https://platform.openai.com/docs/overview (accessed on 1 October 2024)). Globally, the learning objectives are that the students design and build, following the PBL process, a prototype of a system based on the available sensors and actuators in IoTRemoteLab and demonstrate that it works.

**Figure 4:**
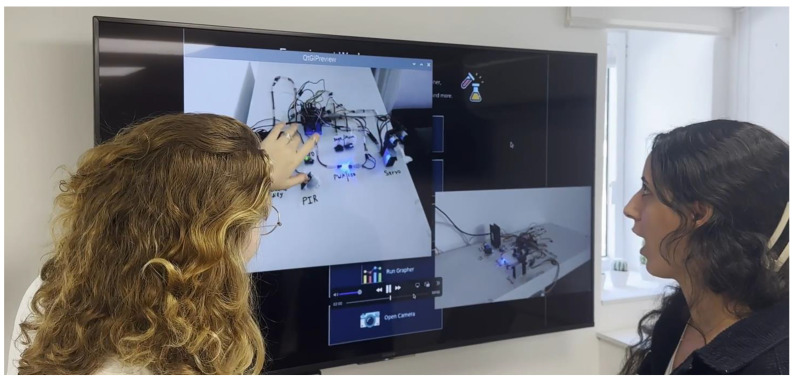
A student touching a screen interfaced with the IoTRemoteLab’s software (v1.0) to interact with the hardware (photo extracted from the video “IoTRemoteLab” [40]).

**Figure 5:**
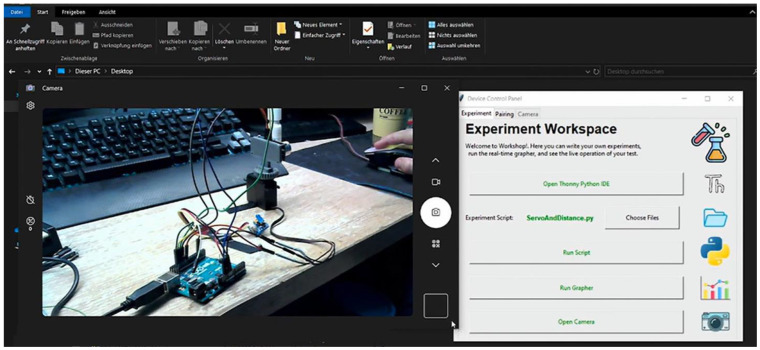
A screenshot of the IoTRemoteLab’s software (v1.0)showing the IoT hardware used for an experiment in real-time (photo extracted from the video “IoTRemoteLab” [40]).

The IoTRemoteLab environment is presented in a video (https://vimeo.com/994422593/ce99c1e5e8 (accessed on 1 October 2024)) [40] that highlights relevant aspects for lecturers, enabling them to create, manage, and enact activities in the lab, illustrating the functionalities available to students using it (Figure 4, Figure 5).

## 4. IoTRemoteLab’s Evaluation

### 4.1. Overview of Results from Questionnaires Answered by Students

Following the design, development, and deployment of IoTRemoteLab, we conducted an evaluation involving 12 undergraduate students enrolled in an elective IoT course offered by the Faculty of Industrial Engineering and Management at the Holon Institute of Technology (HIT), selected based on their high academic performance in automation and industrial robotics courses and their motivation for advanced studies in hardware-related fields. This number of students in the evaluation allows hitting the qualitative research “reliability zone” and obtaining reliable insights [41,42].

The questionnaire we developed was designed to explore various perspectives and gain a comprehensive understanding of IoTRemoteLab’s usability. This allowed us to assess whether the platform effectively facilitates learning, provides an immersive IoT experience, and ultimately leads to a positive and satisfying user journey.

Even though this questionnaire is a non-standard usability one, it is designed to gain insights into specific aspects of IoTRemoteLab’s effectiveness. By collecting feedback from multiple viewpoints, we aimed to determine whether the platform achieves its goals of enhancing learning, creating a realistic IoT experience, and fostering overall user satisfaction.

Each student was introduced to IoTRemoteLab’s environment and provided comprehensive instructions on its usage and purpose, gaining practical experience through hands-on use. The participants were predominantly male (67%, 8/12), with most enrolled in a flexible study track that combined evening and weekend courses year-round (92.5%, 11/12). In Table 1, we present selected items from the questionnaire that highlight key aspects of the educational experience with IoTRemoteLab.

This feedback emphasized the importance of learning programming and coding when using the environment, as these skills are crucial for gaining proficiency in IoT tools and understanding the IoT domain. All respondents also stressed the ongoing need to utilize hardware and associated equipment throughout the course, translating into conducting weekly experiments.

The questionnaire questions examined the efficiency of conducting experiments, the structuring of lesson plans, the impact of the UI on laboratory experiments, the adequacy of the learning process, and how well the lecture process aligned with students’ ideal approaches to an IoT course. This targeted analysis provided valuable insights into IoTRemoteLab’s operational effectiveness and pedagogical alignment, offering a deep understanding of its impact on student learning and the overall success of the IoT workshop.

Moreover, during their experience with IoTRemoteLab, the students reported a significant increase in engagement, attributing it to the lab’s ability to create immersive, interactive environments that effectively bridge the gap between theory and practice through real-time data and simulations.

### 4.2. Students’ Insights-Based Deployment Guided by Lecturers’ Requirements

Below, we analyze the insights emerging from students’ questionnaire responses in light of the lecturers’ requirements guiding our design and development efforts.

The analysis of student feedback to the questionnaire following their IoTRemoteLab experience reveals that their experiences align in many aspects with the requirements set forth by the lecturers and the architectural design of IoTRemoteLab. The students overwhelmingly expressed high satisfaction with the overall experience of the IoTRemoteLab session, with most rating it at 4 or 5 on a 5-point scale. This aligns with the lecturers’ requirement for the lab to provide an engaging and effective learning environment that merges theoretical knowledge with practical application. The balance between theoretical and practical content was particularly well received, confirming IoTRemoteLab’s success in offering a comprehensive educational experience that meets the diverse needs of students.

Several students highlighted the practical components of the workshop as the most enjoyable aspect. This feedback underscores IoTRemoteLab’s ability to facilitate hands-on experiences, a core requirement emphasized by the lecturers. IoTRemoteLab’s architecture, which integrates IoT technologies, was designed to allow students to engage in remote experimentation. This capability effectively bridges the gap between physical and virtual learning environments, enabling students to manipulate and interact with real-world systems from a distance.

While some students faced initial challenges, particularly with the practical aspects, they overcame these with the support provided during the workshop. This points to IoTRemoteLab’s design, which includes a robust UI and support systems that minimize operational failures and ensure smooth communication between the remote infrastructure and the user. The inclusion of real-time feedback mechanisms and intuitive control interfaces, as specified by the lecturers, played a significant role in effectively addressing these challenges.

Some students suggested creating additional communication channels, such as WhatsApp or Telegram groups, to enhance interaction and support. This suggestion aligns with the lecturers’ requirement that the lab support collaborative learning and provide continuous access to resources and assistance. The lab’s architecture, which already supports real-time data sharing and collaborative tools, could be further enhanced by integrating these suggested communication platforms to improve the overall user experience.

Accordingly, the students’ insights from the questionnaire strongly align with the lecturers’ requirements and the architectural goals of IoTRemoteLab. The lab successfully provided a flexible, engaging, and practical learning environment that met the expectations of both students and educators, demonstrating its effectiveness as a tool for modern engineering education.

## 5. Discussion

### 5.1. Overview

This study focused on testing and characterizing a solution for establishing online classes for knowledge acquisition and facilitating lab activities. Insights and conclusions were drawn from lecturers and students as stakeholders. This research also examined technologies that could enhance online lesson plans and explore innovative implementation possibilities in the post-COVID-19 era. The developed environment was assessed for readiness and usability, particularly in creating and characterizing a set of online lessons for the IoT course at HIT catering to Faculty of Industry and Technology Management students.

The environment’s characteristics were evaluated based on perceived benefits, ease of use, user attitudes, and external attributes. These factors were examined to determine their direct or indirect influence on users’ intentions to use the environment. The research process was thorough, encompassing the presentation of alternatives, analyses, risk assessments, and cost evaluations. After filtering various options based on the environment’s needs and requirements, the most suitable alternative was selected.

This study comprehensively explored the problem space, including research questions, objectives, a literature review, and environment specification. It also covered the investigation of the solution space, where different alternatives were presented, and their requirements assessed. This process included meticulously evaluating the most relevant and viable alternative.

Additionally, remote access to the laboratory was tested under international conditions as part of the IDEATION (https://ideation-project.eu/; https://eit-hei.eu/projects/ideation/ (accessed on 1 October 2024)) and DEETECHTIVE (https://www.deetechtive.eu/; https://eit-hei.eu/projects/deetechtive/ (accessed on 1 October 2024)) projects. More specifically, partners from Argentina (DB) and from Europe, all located at the same time in Poland (DKV, MR, JH, MC, MM, JFGG, MB, AS, GB, MW), ran IoTRemoteLab’s software (v1.0) as lecturers and with students from different HEIs involved in IDEATION and DETECHTIVE, while a team at HIT was monitoring the behavior of the physical components remotely used for teaching activities [43].

### 5.2. Strengths and Limitations

We provide an insightful perspective on the advantages and constraints of IoTRemoteLab’s TEL-oriented integrated model. Our goal is to present a comprehensive understanding of the potential benefits and challenges associated with this transformative approach to education.

#### 5.2.1. Strengths

Enhanced accessibility and flexibility: The design and deployment of IoTRemoteLab aim to overcome geographical and physical limitations. By leveraging remote access technologies, students can interact with complex systems and equipment from any location, ensuring continuity in education during disruptions, such as the pandemic, and democratizing access to high-quality engineering education. This initiative aligns with the growing demand for educational models that accommodate diverse learning needs and schedules, particularly relevant in today’s globalized educational context [44].

Student engagement and learning efficacy: Feedback from participants indicates that integrating TEL within the digital twin framework has significantly enhanced student engagement. The immersive remote environments, powered by real-time data and simulations, offer students hands-on experience with industrial applications, effectively bridging the gap between theoretical knowledge and practical application. The ability to manipulate and interact with digital replicas of physical systems has made learning more intuitive and contextually relevant.

Readiness for Industry 4.0: A key outcome of this research is the noticeable improvement in students’ readiness to meet the challenges of Industry 4.0. The laboratory environment, designed around the core principles of IoT and supported by digital twins, provides students with practical experience in smart technologies, automation, and data-driven decision-making. This experience is invaluable in an era where such skills are increasingly essential in the workplace.

#### 5.2.2. Limitations

Despite the strengths highlighted above, several challenges persist. While beneficial in many aspects, reliance on technology introduces potential biases and dependency issues. The performance of the technology, and consequently the learning outcomes, can be influenced by the quality of the simulations and the accuracy of the data models used. Additionally, the initial costs and ongoing maintenance of such advanced systems pose significant financial challenges for HEIs, particularly when balancing these costs with the accuracy and reliability of low-cost components.

The assessment process may not have fully considered all possible user perspectives or unforeseen challenges. The perceived benefits and ease of use might not entirely align with the user experience. External attributes may also fail to encompass all relevant factors impacting the system’s success. The research process may have limitations regarding the scope of the literature review and the comprehensiveness of the analyses and evaluations. Furthermore, selecting the most suitable alternative may not have accounted for all potential risks or unanticipated developments.

#### 5.2.3. Differences with Prior Research

Our research dealing with IoTRemoteLab relates to previous studies on remote labs as a part of STEM education. While several studies have explored this area, focusing on digital twin-based remote laboratories, our work offers a unique perspective by integrating IoT labs into PBL within the HEIs’ context [44]. This approach aligns with the growing emphasis on practical, hands-on learning experiences in STEM and, more specifically, engineering education [45,46].

Furthermore, our work differs from the closely related research by Hussein et al. [44]. This last one deals with a 3D digital twinning simulator (DTS) augmented with VR capabilities, specifically designed for an embedded systems curriculum. In contrast, while both studies utilize remote engineering, our approach delves deeper by integrating TEL and PBL approaches for multidisciplinary curriculum and cross-disciplinarity courses (e.g., automation and robotics, IoT, digital medical technologies). This allows students to apply theoretical knowledge while working on practical projects, fostering a more comprehensive learning experience.

Previous studies, similarly to Hussein’s, highlight the increasing use of VR and augmented reality applications [47,48,49]. The lab we present above is built on TEL principles. Accordingly, it is expected to have additional features and capabilities soon, such as AR, VR, and DTS, after running many experiments that allow collecting real data to build relevant layers for AR and models for VR and DTS.

### 5.3. Future Perspectives

The user perspective on the online class system implementation emphasizes the ease of understanding and using the proposed system. Most participants clearly understood the system’s purpose, indicating a positive user experience. However, additional tools are needed to gather more comprehensive information about the system’s requirements.

#### 5.3.1. Data-Driven Learning: Optimizing Education with Analytics

The presented system will be enriched by incorporating extensive data analytics capabilities to track student engagement and performance and continuous improvement of the teaching methods and the lab environment by developing, for example, DTS. This approach will help maintain and enhance the learning outcomes, aligning with the highest educational and industry standards.

#### 5.3.2. Implementation Perspective

From an implementation standpoint, the focus will be on developing more operational simulations, administering time-to-time questionnaires, and interviewing students and trainees across different study levels over time. Two additional data collection phases are planned to gather more in-depth information. The first phase will involve student simulations with extended questionnaires across various disciplines. The second phase will include publishing and making the software management infrastructure available to HEIs interested in implementing the system, expanding its capabilities, and sharing the new product with the scientific and academic communities. One potential development comprising small robots (such as the Dobot Magician-https://www.dobot-robots.com/products/education/magician.html, available at HIT (accessed on 1 October 2024)) will allow training the students about the challenging tasks to control the actions of distant robots [50,51].

#### 5.3.3. Impact on Education

The anticipated impact on education is expected to be far-reaching, particularly in terms of enhancing the quality of teaching. The introduction of the online and remote-based class system must bring about positive and transformative changes in education, fueling future advancements across a wide spectrum of HEIs and academic research and also on students’ preparedness for new job market positions.

#### 5.3.4. IoTRemoteLab from Education to Research

Looking at HEIs does not consider only teaching; platforms such as IoTRemoteLab must not be limited to this activity. Indeed, another mission of HEIs relies on academic research. Therefore, it is critical to consider remote labs as an opportunity to manage research in a new way, allowing researchers to be involved worldwide. For example, it was demonstrated that a remote, automated control system can significantly support drug or chemical development by allowing collaborative work of disseminated research and optimizing synthesis processes [52,53]. Moreover, remote labs enhanced with AI and cloud computing capabilities, such as “self-driving labs”, can facilitate the discovery of new materials [54,55].

Another challenge in HEIs, both in teaching and research, relies on the emergence and use of knowledge graphs (KGs) or large language models (LLMs). Indeed, dealing with remote labs is today mainly based on predesigned processes such as guided exercises; however, we can expect to have exercises designed on demand by building a particular path in a KG or by asking an LLM to draft dynamically instructions fitting some constraints and educational goals. The approach should resemble current ones used in chemical research, where systems independently (supported by a KG or an LLM) create, plan, and carry out intricate experiments [56,57].

## 6. Conclusions

The successful integration of a lab enhanced by TEL represents a significant shift in engineering education. This study highlights this transformation’s substantial benefits and inherent challenges, particularly concerning accessibility, student engagement, and the practical readiness of engineering and technology students.

This study reaffirms the transformative potential of integrating IoT and TEL in science, technology, and engineering education, emphasizing the dynamic nature of this integration. It advocates for a balanced approach that embraces innovation while critically addressing digital education’s ethical and practical challenges. As this model gains traction, it is poised to set a benchmark for future educational practices, significantly impacting the educational landscape and better preparing students for the technological demands of the modern workforce.

## Figures and Tables

**Table 1:** Descriptive statistics-in-class evaluation of the lab (*MAD*: *median absolute deviation*).

#	Question	Not Agree (1–3)	Agree (4–5)	Median	MAD
1	How do you assess the efficiency and convenience of conducting experiments in the IoT laboratory using the characterized environment?	2	10	4.5	0.5
2	Is categorizing lesson plans into lectures and experiments within the IoT lab appropriately done?	0	12	5.0	0.0
3	To what extent does a user interface that doesn’t necessitate coding for an embedded system enhance the efficiency of laboratory experiments?	8	4	3.0	1.0
4	Do you believe the process of comprehending and gaining knowledge about using the environment and conducting experiments is adequate?	5	7	4.0	1.0
5	How closely does the lecture process using the environment align with your ideal approach to an IoT course?	4	8	4.0	0.0
6	Rate the environment’s compatibility with your learning experience.	1	11	5.0	0.5
7	Is access to all course materials (including experimental results, history, and study materials) convenient and suitable?	2	10	4.5	0.0

## Data Availability

The data presented in this study are available on request from the corresponding author.

## Abbreviations

AI	artificial intelligence
API	application programming interface
DTS	digital twinning simulator
EIT	European Institute of Innovation & Technology
GenAI	generative artificial intelligence
HEI	higher education institution
HIT	Holon Institute of Technology, Israel
HMI	human–machine interfaces
IoT	Internet of Things
KG	knowledge graph
LLM	large language model
PBL	problem-based learning
STEM	science, technology, engineering, and medicine
UI	user interface
VR	virtual reality
TEL	technology-enhanced learning
